# Convolutional Neural Network Model for Intestinal Metaplasia Recognition in Gastric Corpus Using Endoscopic Image Patches

**DOI:** 10.3390/diagnostics14131376

**Published:** 2024-06-28

**Authors:** Irene Ligato, Giorgio De Magistris, Emanuele Dilaghi, Giulio Cozza, Andrea Ciardiello, Francesco Panzuto, Stefano Giagu, Bruno Annibale, Christian Napoli, Gianluca Esposito

**Affiliations:** 1 Department of Medical-Surgical Sciences and Translational Medicine, Sant’Andrea Hospital, Sapienza University of Rome, 00185 Roma, Italy; irene.ligato@uniroma1.it (I.L.); emanuele.dilaghi@uniroma1.it (E.D.); giulio.cozza@uniroma1.it (G.C.); francesco.panzuto@uniroma1.it (F.P.); bruno.annibale@uniroma1.it (B.A.); 2 Department of Computer, Control, and Management Engineering, Sapienza University of Rome, Via Ariosto 25, 00185 Rome, Italy; giorgio.demagistris@uniroma1.it (G.D.M.); cnapoli@diag.uniroma1.it (C.N.); 3 Department of Physics, Sapienza University of Rome, P.le A. Moro 5, 00185 Rome, Italy; andrea.ciardiello@gmail.com (A.C.); stefano.giagu@uniroma1.it (S.G.)

**Keywords:** gastric intestinal metaplasia, virtual chromoendoscopy, BLI, CNN, imaging diagnostics, ResNet50, classification, segmentation

## Abstract

Gastric cancer (GC) is a significant healthcare concern, and the identification of high-risk patients is crucial. Indeed, gastric precancerous conditions present significant diagnostic challenges, particularly early intestinal metaplasia (IM) detection. This study developed a deep learning system to assist in IM detection using image patches from gastric corpus examined using virtual chromoendoscopy in a Western country. Utilizing a retrospective dataset of endoscopic images from Sant’Andrea University Hospital of Rome, collected between January 2020 and December 2023, the system extracted 200 × 200 pixel patches, classifying them with a voting scheme. The specificity and sensitivity on the patch test set were 76% and 72%, respectively. The optimization of a learnable voting scheme on a validation set achieved a specificity of 70% and sensitivity of 100% for entire images. Despite data limitations and the absence of pre-trained models, the system shows promising results for preliminary screening in gastric precancerous condition diagnostics, providing an explainable and robust Artificial Intelligence approach.

## 1. Introduction

Gastric cancer (GC) is the fifth most common neoplasia and the fourth most common cause of death from neoplastic pathology worldwide [1], and its unfavorable prognosis is primarily attributed to late diagnosis at an advanced stage of cancer [2]. However, in low-incidence countries (i.e., European countries), GC screening is not recommended, and the recognition of patients at risk for the development of GC is fundamental to avoiding unnecessary gastroscopies and reducing the burden of care. High-risk patients are those with extensive precancerous gastric conditions or with additional risk factors (i.e., autoimmune gastritis, a family history of GC), and there is evidence that endoscopic surveillance in these patients is beneficial [3]. Intestinal metaplasia (IM) is a gastric precancerous condition characterized by the replacement of the original gastric glands with intestinal epithelium [4]. Gastric IM diagnosis is histological. Therefore, biopsies are necessary during gastroscopy to stage the precancerous conditions and to define the *Helicobacter pylori* status. For reporting the histological evaluation of gastritis and gastric precancerous conditions, the consensus of the updated Sydney system, based on five biopsies of the stomach (two of the antrum and one of the incisura sent in the same vial and two of the corpus sent in another vial), was developed [5]. Therefore, a histological classification system like the Operative Link on Gastric Intestinal Metaplasia (OLGIM) was proposed to stage IM [6]. The OLGIM score includes stages from 0 to IV. According to these studies, patients with advanced stages of OLGIM (such as stages III and IV) have an increased risk of developing GC [6]. Another important risk factor for developing GC is the presence of IM or AG in the context of autoimmune atrophic gastritis (AAG) that extends to the corpus mucosa while sparing the antrum [7]. The topographic extension of IM is important to define, considering that the risk of developing cancerous lesions is closely linked to the extent of IM across the gastric mucosa. In this context, gastric IM can be divided into diffuse or focal, and for this reason, it is necessary to use diagnostic tools for the support and recognition of gastric precancerous conditions during endoscopic examination that allow target biopsies to be performed and patients to be staged correctly. During White Light (WL) endoscopy, random gastric biopsies may fail to detect IM. In recent years, several studies have demonstrated that electronic chromoendoscopy, namely Narrow-Band Imaging (NBI) and Blue-Light Imaging (BLI), has increased accuracy in the diagnosis of precancerous conditions and cancerous lesions [8]. Electronic chromoendoscopy improved the visualization of vascular and mucosal patterns by employing narrower bands of blue and green filters instead of conventional red–green–blue filters [9]. Chromoendoscopically, gastric IM is characterized by some markers, such as the light blue crest, the marginal turbid band, the white opaque substance, and the groove type [10]. The ability to perform target biopsies with electronic chromoendoscopy increases the chance of obtaining a diagnosis of IM and correct staging. In a previous study, NBI showed an accuracy of 85%-90% for the diagnosis of IM and dysplasia [8]. These results were then confirmed in a European multicenter study in which an NBI classification scheme was proposed and validated to stage IM based only on electronic chromoendoscopy appearance (EGGIM—endoscopic grading of gastric intestinal metaplasia). An EGGIM score > 4 has been shown to be associated with advanced stages of OLGIM (III, IV) [11]. Another type of electronic chromoendoscopy (BLI) showed the same results as NBI for gastric IM diagnosis and staging [12]. However, electronic chromoendoscopy is not widespread, and not every endoscopist can use it. Over the past few decades, significant attention has been directed towards the advancement of computer-assisted systems applicable in endoscopy [13]. Computer vision is a crucial field of research in Artificial Intelligence (AI) regarding images and visual data, and convolutional neural networks (CNNs) have completely changed the field of computer vision. CNNs first appeared in 1980 [14]. A CNN is a type of neural network architecture that learns a hierarchy of features directly from a large dataset for a specific task [15]. Several existing image classification algorithms, such as ResNet50 [16], SE-ResNet50, VGG 16, VGG 19 [17], and Inception V3 [18], are used to resolve complex problems related to image classification and segmentation benchmarks. In the task of image classification, the ResNet-50 model appears to be the most well-known and effective architecture in the medical field [19].

However, the development of a CNN for the task of classifying endoscopic images must be based on several factors [20]:

The dataset being retrospective or prospective;
The dataset being based on images or videos;
The dataset being derived from a single center (internal set) or multiple centers (external set);
The use of endoscopic images exclusively in conventional WL or exclusively with electronic chromoendoscopy (BLI, NBI, LCI), or both types of lights combined;
Types of deep learning algorithms (which can include image classification algorithms, object detection algorithms, semantic segmentation algorithms, or a combination of these algorithms).

The task of the CNN can be focused exclusively on a single task (i.e., the recognition of a single precancerous condition, such as intestinal metaplasia) or address multiple tasks (i.e., the simultaneous recognition of IM, AG, and *H. pylori* gastritis).

Different studies have applied AI models for the detection of upper GI lesions, and specifically, GC, especially in Eastern countries, and a recent meta-analysis showed promising results in the application of AI for GC [21,22,23]. Another recent meta-analysis demonstrated good results on the accuracy of AI and its application for early GC diagnosis [24], a condition with a missing lesion rate of up to 20% in Western countries [25].

On this topic, most studies derive from Asian countries, where there is a high prevalence of GC. Conversely, regarding gastric precancerous conditions, only a few studies have focused on the use of AI for AG and IM, and they are exclusively Asian studies, except for a German study on AG [26]. Regarding gastric precancerous conditions, there are three relevant meta-analyses: one about the use of AI for gastric precancerous conditions (based on nine studies) and one about *H. pylori* infection (based on four studies), which have demonstrated a pooled diagnosis accuracy of 90% and 80%, respectively [27]. Another meta-analysis focused on the diagnostic value of AI-assisted endoscopy for chronic AG (based on eight studies) reported a diagnostic accuracy of 98% [28].

Lastly, a meta-analysis was conducted on the diagnostic accuracy of AI exclusively for gastric IM based on 12 eastern studies, demonstrating a pooled sensitivity of 94% and a specificity of 93%. A clinically significant finding of this meta-analysis is the comparison between AI and endoscopists, revealing that AI exhibited higher sensitivity (95% vs. 79%) [29].

The results showed that AI performed excellently in diagnosing GIM, AG, and *H. pylori* infection.

Nonetheless, all the meta-analyses described are based on few studies. Furthermore, there are no Western studies in this field, and developing a CNN system would be useful in supporting endoscopists to recognize and stage IM in a Western country.

For these reasons, this pilot study aimed to develop and assess the precision and recall of a CNN system for the recognition of IM in images of a gastric corpus obtained during gastroscopies with BLI evaluation in a Western country.

## 2. Methods

### 2.1. Participants

We retrospectively collected a dataset of endoscopic images from prospective gastroscopies performed in a single center, Sant’Andrea Hospital, Sapienza University of Rome, from January 2020 to December 2023. Images were obtained during routine weekly endoscopic sessions of outpatients undergoing follow-up for atrophic gastritis corpus-restricted, multifocal, and extensive atrophic gastritis, celiac disease, and anemia.

The exclusion criteria were individuals below 18 years of age, incomplete or unavailable clinical data, insufficient gastric biopsies, contraindications for biopsies, patients who had undergone partial or total gastrectomy, and instances involving neoplastic gastric lesions. Ethical approval and informed consent to collect endoscopic images were obtained from all patients.

### 2.2. Endoscopic and Histological Procedures

Gastroscopies were performed using Fuji scopes with the use of pharyngeal anesthesia (xylocaine spray puffs) and/or conscious or deep sedation (midazolam or propofol). Gastroscopies were performed using High-Resolution (HR) White Light (WL) endoscopy, followed by a BLI assessment. For the aim of this study, multiple images were taken during the BLI assessment of the regions of the antrum and the corpus. If IM was endoscopically observed with the use of BLI, multiple images of the area of interest were taken, and targeted biopsies were performed. During the BLI assessment, if no IM was detected, multiple general images of gastric mucosa were taken, and biopsies according to the updated Sydney system protocol were performed [5]: two biopsies of the antrum, one of the incisura angularis, and two of the corpus. These biopsies were then placed in separate vials for histopathological evaluation, the gold standard for the diagnosis of gastric IM. Additional biopsies were obtained when other abnormalities were identified and subsequently submitted for histopathological examination.

### 2.3. Image Dataset

For each patient included in the study, a data collection form was administered to collect demographic, endoscopic, and histological data. The images were acquired at a single center during dedicated sessions by two experienced endoscopists, thereby minimizing the risk of selection bias. All images of these gastroscopies were collected in a JPEG format, and after image acquisition, a resident endoscopist discarded WL images, esophagus images, duodenum images, and poor-quality images (i.e., out-of-focus or low-resolution images). The criteria for selecting images were the suitability of images to elucidate the presence or absence of IM; adequate graphic quality; accordance between endoscopic and histological evaluation; and guarantee of the anonymity of the images. Images with histological evaluation compatible with pseudopyloric metaplasia were excluded from the dataset.

Subsequently, an expert endoscopist categorized all the remaining gastric corpus and antrum BLI images into IM-positive and IM-negative images. For this study, only gastric body images that were acquired using BLI assessment were used for the CNN model development. Finally, an expert endoscopist of gastric precancerous conditions performed annotations of regions of interest that contained informative features using the Image J software (Image 1.53 t).

### 2.4. Development of AI Models

**Figure 1:**
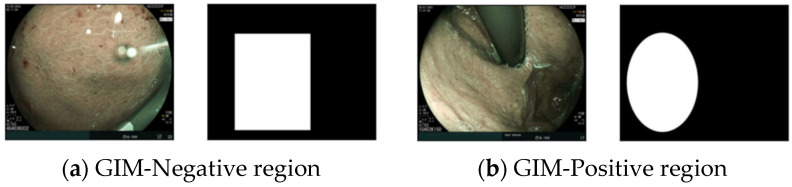
Samples from the dataset showing BLI images with corresponding annotations.

The primary challenges were data availability and annotation quality. Due to the absence of pixel-wise segmentation masks, it was not feasible to tackle the problem as a segmentation task. Instead, we collected annotations on regions of interest, highlighted as rectangular or oval regions in the images, as shown in Figure 1.

**Figure 2:**
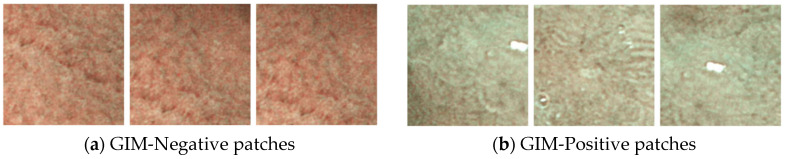
Examples of randomly sampled patches from GlM-negative and GlM-positive regions.

The annotations, labeled as IM-positive or IM-negative, highlight regions with discriminative features (Figure 1). Our approach involves extracting and classifying 20 random patches of 200 × 200 pixels from each annotated region (Figure 2). The data were split into 80% for training, 10% for validation and hyperparameter tuning (Section 4), and 10% for testing. Various CNN architectures were tested, including ResNet50, VGG 16, VGG 19, and Inception V3, with the best results achieved using ResNet50. The models were trained from scratch using the Adam optimizer [30] for 200 epochs, selecting the best model based on validation loss. All images were used for testing once only. We calculated the accuracy, recall, and precision of this model. Recall is a metric used to determine the frequency at which a machine learning model accurately identifies true positive cases among all the actual positive cases in the dataset. It is also known as sensitivity. On the other hand, precision assesses how frequently a machine learning model accurately predicts the positive class. Precision can also be called specificity.

## 3. Results

Overall, 279 patients were included (62.7% female). The histological examination revealed the absence of gastric IM in 146 patients, while 133 patients tested positive for gastric IM. Among those with positive results, 81 cases exhibited IM exclusively in the corpus, with the antrum spared. Regarding the histological staging of IM, 146 patients were categorized as OLGIM 0, 50 as OLGIM I, 63 as OLGIM II, 14 as OLGIM III, and 6 as OLGIM IV. Chromoendoscopic staging of IM using the EGGIM score was performed in 227 patients, with 108 classified as EGGIM 0, 148 as EGGIM 1–4, and 22 as EGGIM >4. Twenty-two patients tested positive in the histological examination for *H. pylori* infection.

The final dataset included 1384 high-resolution BLI images, with 721 gastric corpus images classified as IM-negative and 663 as IM-positive. Each image had a resolution of 1279 × 1023 pixels. A total of 1384 images yielded 505 IM-positive and 771 IM-negative annotations. Finally, a total of 6103 IM-positive and 4466 IM-negative patches were obtained. Each image patch had a resolution of 200 × 200 pixels. We split the dataset into three phases: the training set (which contained 4861 positive patches and 3403 negative patches), the validation set (which contained 681 positive patches and 521 negative patches), and the test set (which contained 561 positive patches and 542 negative patches). Images were saved in JPEG format and are available upon reasonable request to the corresponding author.

The model’s performance on the patch test set, with patches classified as either IM-positive or IM-negative, demonstrated an accuracy of 74%, a precision of 76%, and a recall of 72%, as illustrated in Table 1. To support the statistical significance of these results, we performed a significance test comparing the performance of ResNet-50 with other CNN architectures like DenseNet, EfficientNet, and XceptionNet. Using a paired *t*-test, we found that ResNet-50’s performance was significantly better than the other architectures (*p* < 0.05).

**Figure 3:**
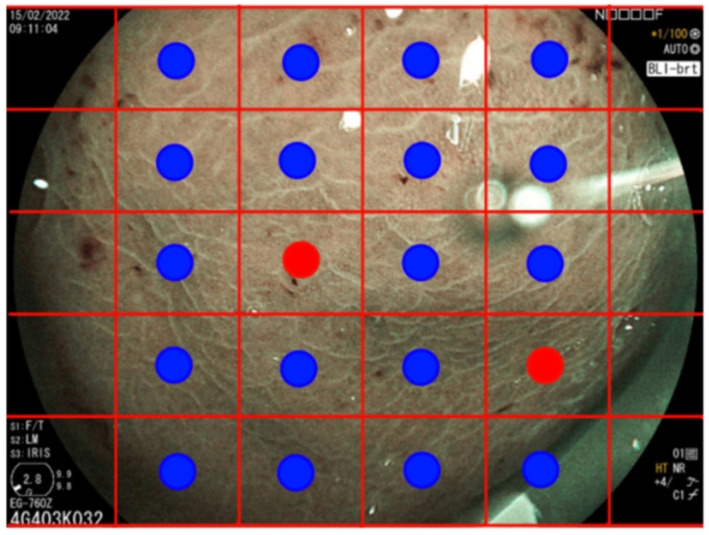
Illustration of the classification algorithm for entire images. The image is divided into 30 non-overlapping patches that are individually classified, followed by a voting scheme to determine the overall image classification.

While the patch-level results revealed certain limitations for standalone diagnostic use, they formed the foundational layer for further enhancements. For the classification of entire images as either IM-positive or IM-negative, we developed a custom voting scheme to achieve this, as illustrated in Figure 3. The voting scheme in our methodology involves learning a decision threshold to classify entire images based on the classification of individual patches. First, each image is divided into 30 non-overlapping patches. Our convolutional neural network (CNN) model, specifically the ResNet-50 architecture, is trained to classify each patch as either positive or negative for intestinal metaplasia (IM). Then, we employ a linear search to determine an optimal threshold for the number of patches that need to be classified as positive for the entire image to be considered positive. This threshold is learned by maximizing the overall image classification accuracy on the validation set. For each image, if the number of patches classified as positive exceeds the learned threshold, the entire image is classified as positive. Conversely, if the number of positive patches is below the threshold, the image is classified as negative. During the validation phase, we iteratively adjusted the threshold and evaluated the performance to ensure that the chosen threshold maximizes the overall accuracy. This process helps in fine-tuning the model to strike a balance between sensitivity (recall) and specificity (precision). Table 2 details the configurations yielding the most effective results. One of the best configurations, with a decision threshold of 0.8 and a patch threshold of 13/30, showed an accuracy of 78%, a precision of 70%, and a recall of 100%. This voting scheme and threshold optimization enhance the robustness and practical applicability of our method, allowing us to achieve high accuracy in classifying entire endoscopic images based on patch-level predictions. Our method enhances explainability and interpretability over traditional CNN classifiers by allowing practitioners to inspect which specific patches influenced the final classification decision. For instance, patches showing strong specular reflections might have been erroneously classified as IM-positive. Such errors were readily identifiable and could be corrected, thereby enabling a more robust and interpretable diagnostic process.

## 4. Discussion

Most studies regarding AI in upper gastrointestinal management have focused on the detection of early gastric cancer [21,22,23,24], while only a few studies, exclusively from Asian countries, have investigated these learning systems for gastric precancerous conditions [27,28,29].

However, given the significant differences in diagnostic outcomes between countries with a high and low prevalence of GC, it is imperative to also investigate a specific CNN for the recognition of GC in countries with a low prevalence of GC. Furthermore, in the Western world, where there is a low prevalence of gastric cancer, recognizing patients with precancerous conditions who will subsequently undergo endoscopic follow-ups is crucial. For these reasons, our CNN could provide valuable support in identifying these conditions. Our system demonstrated high recall and moderate precision, making it particularly suitable for initial screening applications. However, in countries with a low prevalence of gastric cancer, a recognition system with greater precision would be more useful in terms of economic resources. In the initial stages of screening contexts, it is preferable to have high recall in order to have fewer false negatives. The decision to prioritize the identification of IM is based on its pivotal role in the gastric carcinogenic process, marking a critical point of no return. Additionally, in this study, we focused on IM, and distinguishing between IM and pseudopyloric metaplasia is crucial because studies indicated that the latter is not associated with the development of GC [31]. Pseudopyloric metaplasia and other conditions, such as foveolar hyperplasia or exclusive gastric atrophy, could be disturbing factors for the correct functioning of the learning machine and should be investigated in future studies.

A relevant meta-analysis on the diagnostic accuracy of AI regarding gastric IM conducted subgroup analyses to examine factors influencing AI performance in recognizing gastric IM, including the number of images (>1500 or <1500), the study design (prospective or retrospective), the study center (multicenter or single center), the endoscopy type (WL only or others), and the algorithm type (classification algorithm or others). The analysis revealed that the type of algorithm is the most significant factor that significantly impacts precision (also known as specificity) [29]. By comparing our system with previous AI models for IM detection extracted from the meta-analysis already described [29], our study is the only one that uses the ResNet-50 algorithm in this context. We conducted extensive experimentation with convolutional neural network (CNN)-based architectures such as ResNet, DenseNet [32], EfficientNet [33], and XceptionNet [34], each of which consists of a CNN component for feature extraction and a linear classifier for making predictions. The aim was to determine which model performed best on our specific task, single patch classification, where only the regions of interest were considered, while irrelevant regions were ignored. Our comparative analysis focused on several key factors: 1. Performance: Through rigorous testing, we observed that ResNet consistently achieved the highest accuracy on our dataset. This superior performance was crucial in our decision, as accuracy directly impacts the reliability and effectiveness of the classification task. 2. Complexity: ResNet’s architecture, characterized by its residual connections, allows for training deeper networks without the common issue of vanishing gradients. This balance between depth and ease of training made ResNet more suitable for our needs compared to more complex networks like DenseNet and EfficientNet, which, despite their advanced capabilities, did not provide accuracy improvements in our experiments. 3. Suitability for the task: The residual connections in ResNet facilitate better feature extraction from the patches by enabling the network to learn residual mappings, which are particularly effective for distinguishing between the classes in our dataset. This architectural advantage made ResNet more adept at handling the specific nuances of our classification task.

Regarding precision, which in our study was shown to be lower than recall (also called sensitivity), it is in line with the results of most of the previous studies analyzed, which all report values higher than 80% [29]. The diagnostic accuracy of AI in the studies analyzed in this meta-analysis averaged around 90% [29], while our study achieved approximately 80% diagnostic accuracy, although we reported very high recall values of approximately 100%. Comparing our system to earlier AI models for IM detection [29], we found that our system is the only one developed based exclusively on BLI assessment. Most of the previous studies used endoscopic images with electronic chromoendoscopy, such as NBI, BLI, and Linked Color Imaging (LCI), but only one study developed a system using WL, NBI, and BLI [35]. The other systems were developed either with NBI assessment alone or with a combination of WL and NBI assessment.

Another unique aspect, which can also be seen as a limitation, is that our system focuses exclusively on the gastric corpus. However, this is a preliminary analysis, and image collection also involved the antral region, which will be subsequently analyzed for the entire stomach.

To further improve our model, we conducted a detailed error analysis to understand common misclassifications and identify areas for enhancement. One common issue was that some patches containing strong specular reflections or image artifacts were erroneously classified as IM-positive. By implementing preprocessing steps to reduce or eliminate these reflections and artifacts, we could enhance the model’s accuracy. Techniques like glare removal and advanced image normalization could be beneficial. Another challenge we encountered was with patches from out-of-focus or low-quality images, which led to incorrect classifications. Developing a quality assessment module to filter out low-quality patches before classification could prevent such errors. Training a separate CNN to detect and exclude poor-quality patches might improve overall accuracy. Some patches contained regions that were difficult to classify, even for human experts. Enhancing the training dataset with more annotated examples of these ambiguous regions could help the model learn better representations. Rare patterns and edge cases not well represented in the training data were often misclassified. Expanding the dataset to include more examples of rare patterns and edge cases could help the model generalize better. Collaborating with multiple medical centers to gather a more diverse dataset would be advantageous.

Based on the error analysis, several potential improvements were identified to enhance our model’s performance. Implementing advanced preprocessing techniques such as glare removal, noise reduction, and image normalization could improve patch quality and reduce misclassification due to artifacts. Incorporating a quality assessment module to automatically detect and exclude low-quality patches before classification could enhance overall accuracy. Collaborating with additional medical centers to gather a more diverse and representative dataset, including rare patterns and edge cases, could improve the model’s ability to generalize to new and unseen data. Developing a multistage classification pipeline where an initial model filters out obvious negatives, followed by a more detailed analysis of potential positives, could reduce false positives and improve precision.

By conducting this study, we identified significant issues. Many of the images that were acquired during the endoscopic examination are of poor quality. Images with disturbing factors such as mucus, bubbles, or reduced visibility were discarded for CNN deep learning creation. To reduce the presence of mucus and bubbles in the gastric mucosa, there are studies that suggest premedication before the endoscopic examination [36]. This approach could enhance image quality and reduce the time spent washing/cleaning the gastric mucosa, allowing for more time to detect cancerous and precancerous conditions. Another important point is that the detection of gastric precancerous conditions during gastroscopy must be based on a high-quality gastroscopy, with specific performance measures for upper gastrointestinal endoscopy (exploration time, taking standardized photos, routine use of electronic chromoendoscopy, etc.) explained in the guidelines [37]. In this regard, it would be interesting in future research to explore the development of AI to enhance the quality control of upper gastrointestinal endoscopy [38]. Another problem is image storage spaces that allow for manipulation (such as the annotations of images), always considering that they are sensitive data obtained following authorization expressed in the informed consent of the endoscopic examination. Furthermore, our study had some limitations. Our dataset had a limited number of images, though there is no mandatory minimum number of images for accurate deep learning development. Previous studies used datasets ranging from 84 to 17,000 images to train deep learning models, showing no differences in diagnostic accuracy outcomes [29]. The development of deep learning was only carried out for the gastric corpus. The development of a CNN that investigates only one region of the stomach cannot be used in real practice in the general population but could be useful in identifying the percentage of patients at high risk of developing GC, such as patients with autoimmune gastritis [7]. To date, the gold standard for autoimmune gastritis diagnosis is the histopathological assessment by gastric biopsies during gastroscopy, although non-invasive serological screening before endoscopy may offer utility in some clinical contexts (e.g., serum biomarkers like parietal cells and intrinsic factor, autoantibodies, or serum gastrin and pepsinogens). According to the guidelines, patients with autoimmune gastritis with the presence of AG and/or IM that extends to the corpus mucosa while sparing the antrum require a surveillance gastroscopy every 3 years [3].

However, we will aim to also extend this methodology to the gastric antrum region, enhancing the utility and applicability of our diagnostic tool. Additionally, we will plan to improve the precision and accuracy of our system through semi-supervised pretraining strategies.

Using a dataset comprising thousands of gastric mucosa endoscopic images in WL for pre-training the system, increasing the number of images by including the antral region, and increasing the patient pool are all strategies that could enhance the performance of the CNN system. These strategies are particularly advantageous as they do not require a large, labeled dataset, thus overcoming one of the significant hurdles in medical image analysis. However, for data augmentation, we have already used techniques such as vertical/horizontal reflections, random rotations, and uniform scaling.

An innovative methodology in regenerative medicine to expand the dataset is the assessment of hyperspectral imaging and CycleGAN-simulated data, which have already shown promising results in the field of upper gastrointestinal endoscopy for the detection of early esophageal cancer [39]. Although all existing studies are in the early stages of development with only internal validation [40], this is an emerging field in medical AI. Research in this area is expected to grow exponentially in the coming years and should be considered for future studies. Our efforts will focus on refining these techniques to provide more reliable and robust diagnostic solutions.

The implementation of CNN models in routine endoscopic practice will enhance the quality of gastroscopy by improving the detection of patients at risk for the development of gastric cancer (i.e., those requiring endoscopic surveillance) as well as healthy patients. In this last subgroup of patients, AI assistance will potentially eliminate the need for biopsies on healthy mucosa, reducing the burden and healthcare costs involved in endoscopy. Additionally, more accurate detection of precancerous conditions will help reduce unnecessary follow-up gastroscopies.

By addressing these areas for improvement, we aim to enhance the accuracy and reliability of our deep learning system for detecting gastric IM.

In conclusion, in this pilot study, we have introduced a novel AI-driven approach for the diagnosis of gastric IM in a Western country and constructed a CNN system using endoscopic image patches from a monocentric hospital that was able to achieve high recall/sensibility and thus a low number of false negatives for the detection of IM in the gastric corpus.

## Figures and Tables

**Table 1:** Performance metrics of the CNN model on the patch test set, with patches classified as either GIM-positive or GIM-negative.

Model	Accuracy	Precision	Recall
ResNet	74%	76%	72%

**Table 2:** Test set metrics for entire image classification, showing the best configurations of decision and patch thresholds.

Decision Threshold	Patch Threshold	Test Accuracy	Test Precision	Test Recall
0.5	23/30	78%	75%	81%
0.8	13/30	78%	70%	100%
0.5	24/30	76%	68%	83%

## Data Availability

The data presented in this study are available on request from the corresponding author due to privacy.
